# Odontogenic Infection Associated with Facial Vascular Malformation: Diagnostic, Surgical, and Quality-of-Life Considerations That Should Not Be Overlooked

**DOI:** 10.3390/jcm15072721

**Published:** 2026-04-03

**Authors:** Kamil Nelke, Klaudiusz Łuczak, Michał Gontarz, Angela Rosa Caso, Maciej Janeczek, Ömer Uranbey, Dayel Gerardo Rosales Díaz Mirón, Maciej Dobrzyński, Małgorzata Tarnowska, Piotr Kuropka

**Affiliations:** 1 Maxillo-Facial Surgery Ward, EMC Hospital, Pilczycka 144 Street, 54-144 Wrocław, Poland; 2 Academy of Applied Sciences, Health Department, Academy of Silesius in Wałbrzych, Zamkowa 4 Street, 58-300 Wałbrzych, Poland; 3 Department of Cranio-Maxillofacial Surgery, Jagiellonian University Medical College, Jakubowskiego 2 Street, 30-688 Cracow, Poland; 4 Department of Oral and Maxillo-Facial Surgery, University of Siena, Viale Aldo Moro, 2, 53100 Siena, SI, Italy; 5 Department of Biostructure and Animal Physiology, Wrocław University of Environmental and Life Sciences, Cypriana K. Norwida 31 Street, 50-375 Wrocław, Poland; 6 Department of Oral and Maxillofacial Surgery, Faculty of Dentistry, Aydın Adnan Menderes University, Aydın 09100, Türkiye; eomeruranbey@gmail.com; 7 Instituto de Seguridad y Servicios Sociales de los Trabajadores del Estado (ISSSTE) Hospital Regional de Torreon, Hospital Regional de Torreón, Torreón 27000, Mexico; cmfdayel@gmail.com; 8 Department of Pediatric Dentistry and Preclinical Dentistry, Wrocław Medical University, Krakowska 26 Street, 50-425 Wrocław, Poland; 9 Division of Histology and Embryology, Department of Biostructure and Animal Physiology, Wrocław University of Environmental and Life Sciences, Cypriana K. Norwida 25, 50-375 Wrocław, Poland

**Keywords:** Hemangioma, facial deformation, vascular lesion, jaw cyst, odontogenic infection

## Abstract

**Background and Clinical Significance**: Vascular lesions of the face, particularly arteriovenous malformations (AVM) and mixed hemangiomas (MH), pose significant diagnostic and therapeutic challenges because of their complex anatomy, unpredictable behavior, and high risk of bleeding. Surgical planning should be individualized and often requires a staged approach with meticulous interdisciplinary coordination to ensure patient safety. The presence of a concomitant odontogenic infection further complicates management, as local inflammation may exacerbate vascular instability and increase the risk of life-threatening complications. Local inflammation and infection might cause some life-threatening conditions, especially when an abscess occurs in the area of any vascular lesion. Ensuring that the oral cavity is free from potential odontogenic infections is a particularly important issue in many complex cases, especially in patients treated for oral, head, and neck cancer or in those with other coexisting morbidities affecting the oral and facial regions. **Case Presentation**: A 72-year-old man was referred for management of a severe odontogenic infection associated with an extensive facial vascular lesion. The patient’s medical history was significant for arterial hypertension and chronic liver dysfunction (CLD) of unclear etiology. Complete blood testing, including coagulation assessment and liver ultrasonography, was performed, with no contraindication to surgery identified. The scope of odontogenic-related infections was scheduled for simultaneous removal during initial surgery. Preparation for surgery included the local application of sclerotherapy agents. **Conclusions**: Quite often, a routine panoramic radiograph can help in assessing the status of bone and dentition to undertake all necessary treatment. Severe odontogenic disease, including multiple retained roots, periapical infections, and odontogenic cystic lesions in the context of poor oral hygiene, may lead to the occurrence of possible inflammation. In case of any vascular lesion, a careful diagnostic and therapeutic strategy is needed. This case report highlights that maintaining an infection-free oral environment is a critical component of care in patients with complex facial MH and should be regarded as an essential element of overall treatment planning.

## 1. Introduction

**Figure 1:**
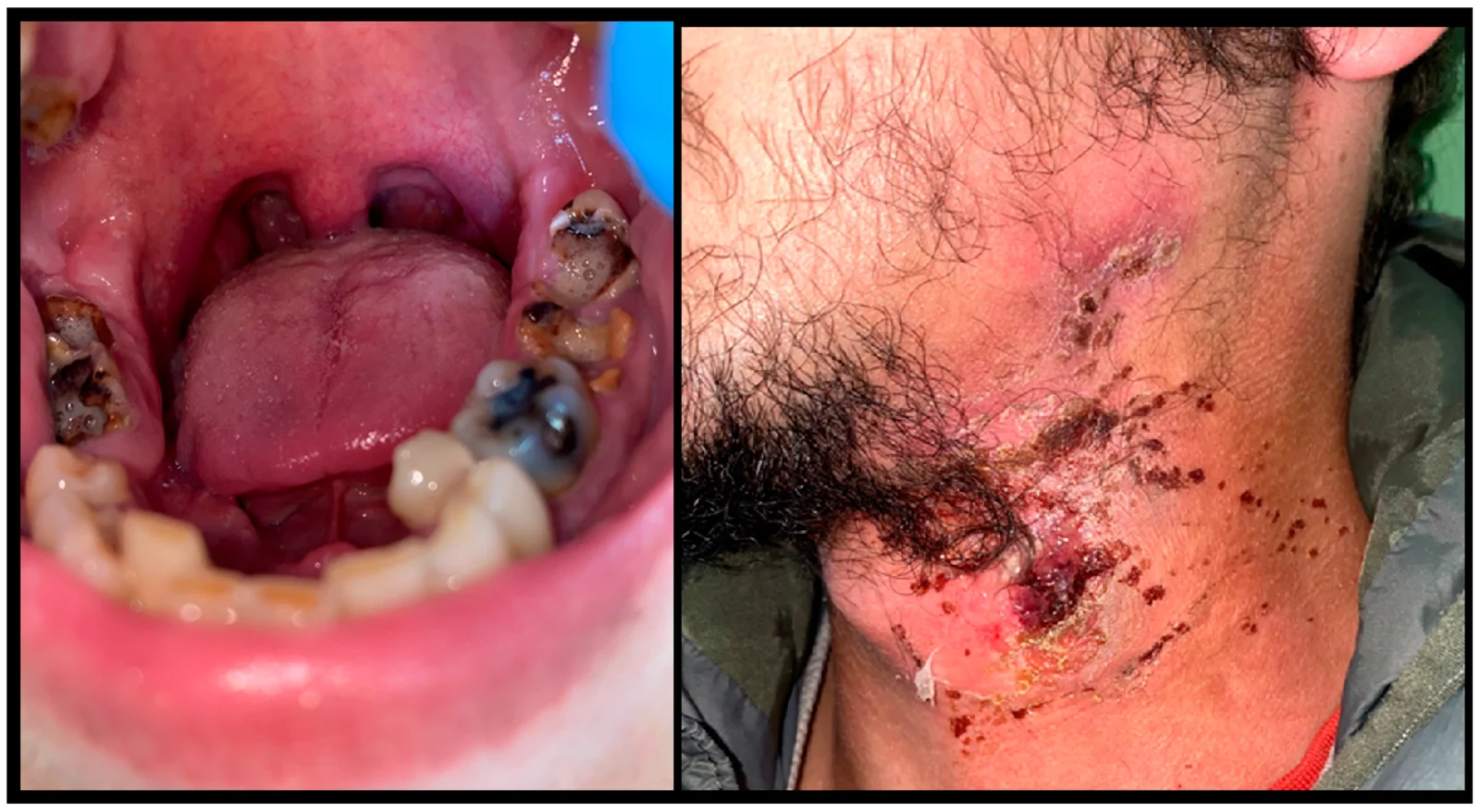
An example of a case with a lot of teeth-related (odontogenic) sources of inflammation causing visible submandibular swelling, abscess formation with lymphonodulitis, skin irritation and very poor overall oral, skin and facial condition. A quite significant problem arises if a similar source of inflammation is found in a patient with advanced facial vascular lesions.

The oral and maxillofacial region comprises a wide spectrum of pathological conditions, among which odontogenic lesions are particularly prevalent [1]. The presence of active, chronic, or acute odontogenic infection, including sinus-related manifestations such as odontogenic sinusitis, represents a significant clinical problem in oral and maxillofacial practice [2]. In many cases, typical clinical investigation of such condition is greatly limited and improved diagnostics with CBCT (cone beam-computed tomography) is constantly increasing the odds of identifying possible sources of local infection. Odontogenic infections arise primarily from dental caries, periodontal disease, periapical pathology, or retained roots and, if left untreated, may spread through fascial planes, bone marrow spaces, or vascular pathways. These infections can progress from localized inflammatory lesions to extensive regional involvement, potentially affecting adjacent anatomical structures such as the maxillary sinus, submandibular space, or deep cervical compartments [3]. In advanced cases, untreated dental infections may lead to severe complications, including cellulitis, abscess formation, osteomyelitis, septic thrombophlebitis, airway compromise, or systemic sepsis, all of which may become life-threatening without timely diagnosis and intervention [4]. When sources of such infection are present in the cases of patients after head and neck radiotherapy, those who also have facial vascular lesions and are undergoing any medication treatment (like bisphosphonates or denosumab uptake) will see a significantly increased risk of serious complication. The risk is particularly elevated in elderly patients or those with systemic comorbidities, impaired immunity, or coexisting pathological conditions in the craniofacial region [5]. The early identification and elimination of odontogenic foci are therefore essential components of comprehensive patient management and the prevention of serious complications. Figure 1 demonstrates the potential progression of untreated odontogenic infections to abscess formation, purulent fistula development, lymphadenitis, and localized mandibular osteomyelitis (Figure 1).

Vascular anomalies (VAs) of the head and neck encompass a heterogeneous group of congenital lesions that include hemangiomas (HAs) and vascular malformations (VMs), such as arteriovenous malformations (AVMs). These conditions are characterized by complex anatomical relationships, variable clinical behavior, and the potential for severe complications, including massive hemorrhage, tissue destruction, and functional impairment. When located in the facial and maxillofacial region, vascular lesions represent a significant diagnostic and therapeutic challenge, requiring meticulous evaluation and individualized treatment planning [6,7,8,9]. Many local and general factors can influence the VA’s shape, size and growth; it might even cause the occurrence of some worrisome bleeding if not addressed properly, if one of those factors are related to local sites of dental inflammation.

According to the Mulliken and Young classification, vascular lesions are divided into hemangiomas most commonly observed in early infancy and VMs, which frequently affect the maxillofacial region and may present as slow-flow, fast-flow, or mixed lesions, where a mixed hemangioma (MH) is one of them [10,11]. Maxillomandibular vascular lesions may exhibit unpredictable growth patterns, variable anatomical involvement, and complex hemodynamics; therefore, extensive lesions should be managed in specialized centers with expertise in vascular anomalies. These lesions are potentially life-threatening due to their propensity for spontaneous bleeding or hemorrhage triggered by routine procedures such as tooth extraction, local anesthetic injection, or minor dental interventions [10,11,12,13]. Management strategies include both surgical and non-surgical approaches, such as electrocoagulation, sclerotherapy, intravascular embolization, and conservative, radical, or palliative surgical resection. Many treatment protocols involve staged procedures, with palliative strategies often aimed at improving quality of life (QOL), preventing bleeding, and restoring essential functions such as speech, mastication, swallowing, and breathing [12,13,14,15,16,17,18].

Mixed vascular lesions, exhibiting features of both hemangiomas and vascular components, may simultaneously involve bone, soft tissues, and neurovascular structures. Their management typically requires a multidisciplinary approach, integrating radiological assessment, surgical expertise, and careful perioperative planning. The risk of uncontrollable bleeding remains one of the most critical concerns, particularly when invasive procedures are contemplated. In patients with extensive facial vascular anomalies, the dental component of disease is not limited to infection alone. Progressive caries, retained roots, periodontal breakdown, traumatic occlusion, and difficulty maintaining oral hygiene may create a persistent inflammatory burden while simultaneously restricting safe access to routine dental treatment. As a result, these patients may experience chronic pain, halitosis, impaired mastication, reduced oral intake, recurrent mucosal trauma, and deterioration in oral health-related quality of life [10,11]. A major problem arises when local dental infections involve the vascular lesion or when abscess formation is present.

Odontogenic infections are among the most common inflammatory conditions in the maxillofacial region. Although they are generally manageable in otherwise healthy individuals, their presence in patients with facial vascular anomalies introduces substantial additional risk. Local inflammation may increase tissue vascularity, compromise hemostasis, and facilitate the spread of infection to deep anatomical spaces. In the setting of an underlying vascular lesion, these factors may precipitate rapid clinical deterioration and, in extreme cases, life-threatening events [12,13,14,15].

Ensuring adequate oral health and controlling odontogenic sources of infection is therefore a critical, yet often underestimated, component of care in patients with facial vascular lesions. The assessment of dentition, periapical status, and jawbone integrity is essential during preoperative planning, not only for vascular anomalies but also for patients undergoing treatment for head and neck malignancies or other complex maxillofacial conditions. Although panoramic radiography often provides valuable initial information, diagnostic evaluation may be limited by anatomical distortion or overlapping structures, necessitating advanced imaging modalities, such as cone beam-computed tomography (CBCT) or a classic CT. Improved diagnostics, combined with accurate patient examination, can help determine the scope of necessary surgical, dental, and local interventions required to enhance the condition of the jawbones and oral cavity [10,11,12,13,14,15,16,17,18,19,20].

Despite its clinical relevance, the interaction between facial vascular anomalies and odontogenic disease remains sparsely addressed in the literature. Most reports focus primarily on the vascular lesion itself, with limited discussion of how concomitant dental pathology influences treatment decisions, timing, and overall risk. As a result, the importance of early dental assessment and infection control may be underestimated [16,17,18,19,21].

The present report describes a complex case of an MH facial lesion involving the mandible and adjacent soft tissues, complicated by severe chronic odontogenic disease, odontogenic cysts, periapical lesions, and odontogenic sinusitis. Through this case, we aim to emphasize the diagnostic challenges, surgical considerations, and the critical role of oral infection management in ensuring patient safety and optimizing outcomes.

## 2. Case Presentation

**Figure 2:**
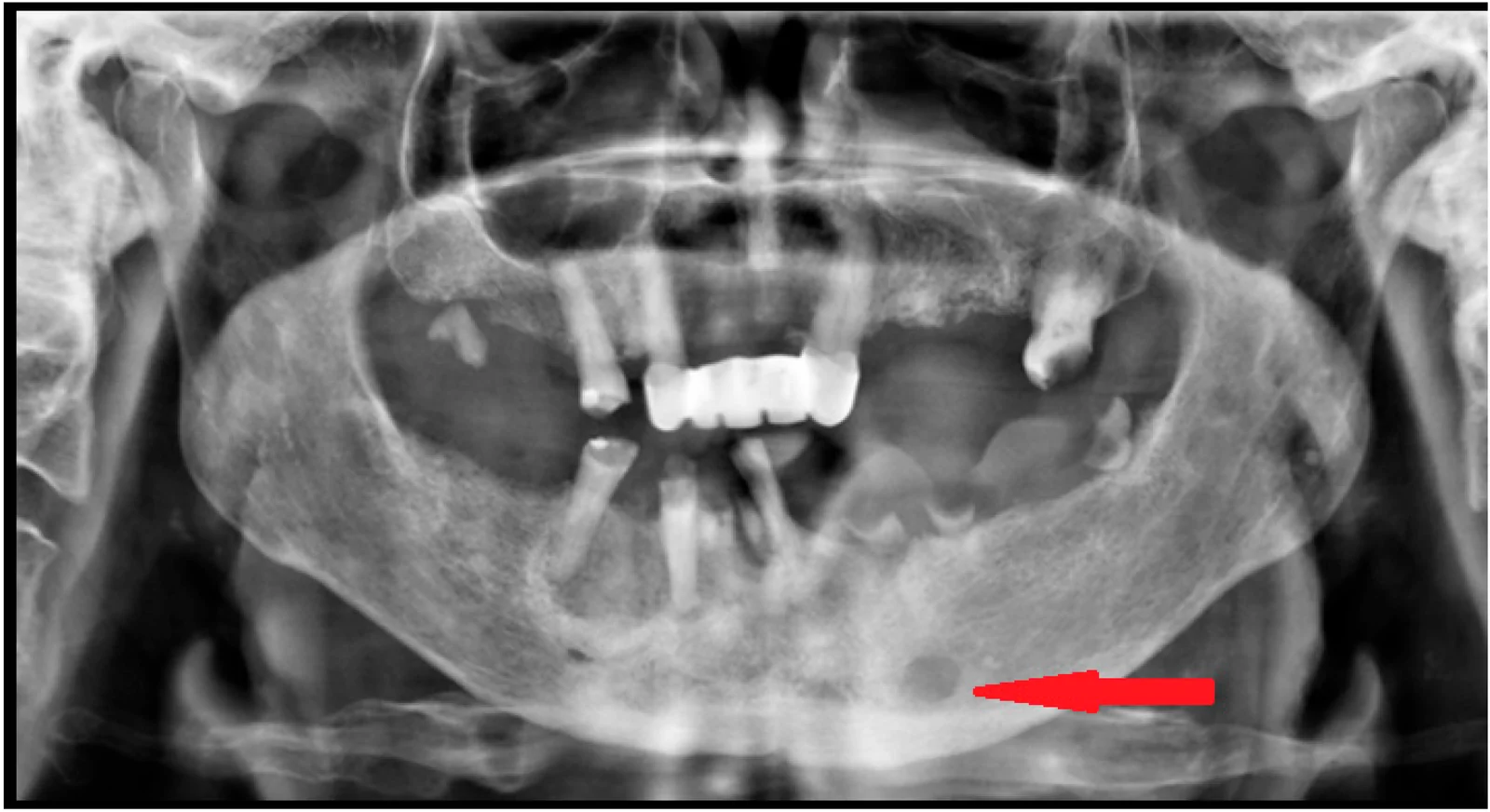
Local dental infections caused by various status of teeth, retained dental roots, periapical changes, periodontitis and active odontogenic infection might have different spectra and manifestations over time. Odontogenic sinusitis (ODS) is quite commonly caused by decayed teeth and occurrence of periapical lesions in some teeth. A 72-year-old patient treated for hypertension, some liver function disorders and who had a massive left-sided facial hemangioma.

**Figure 3:**
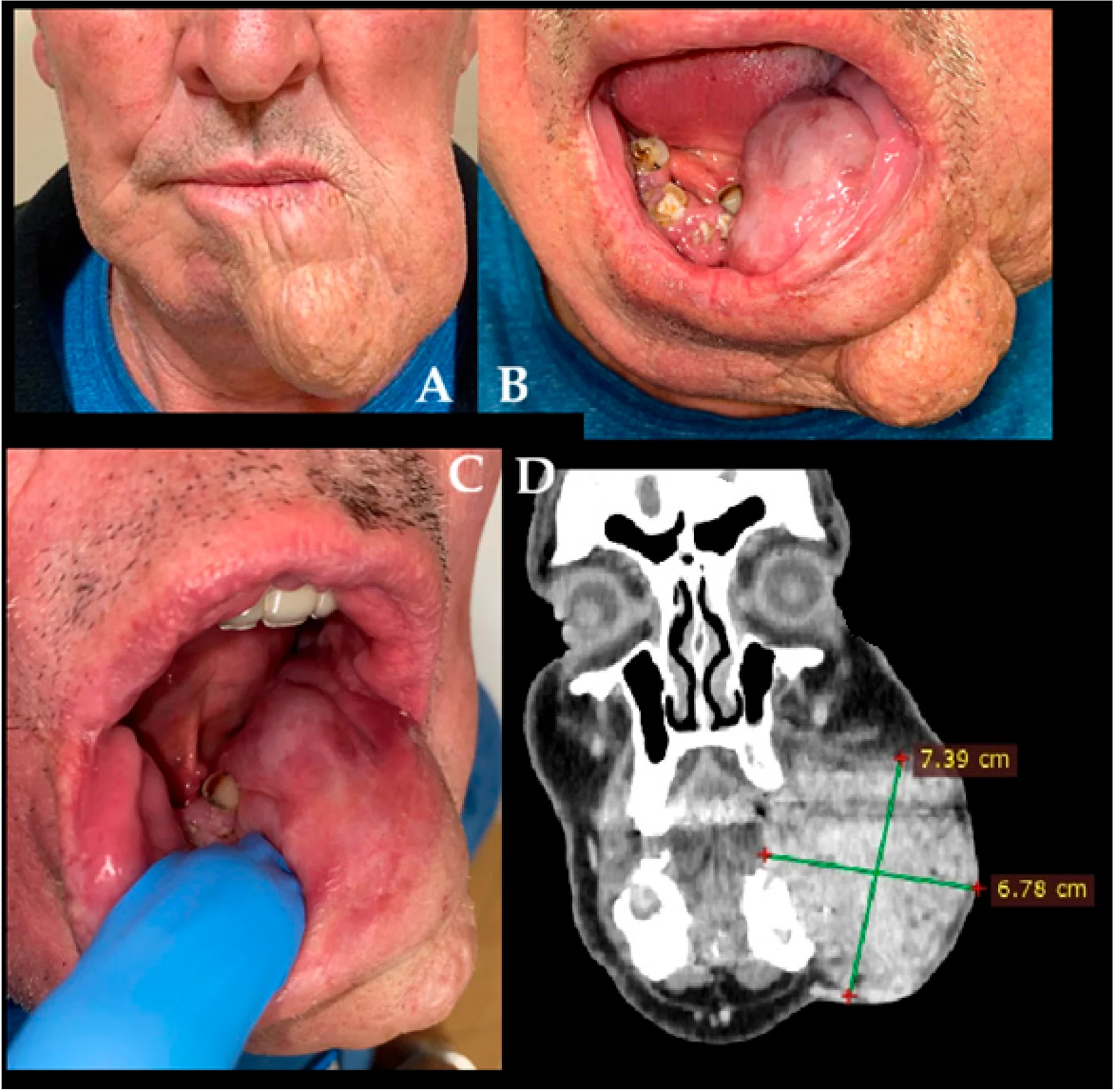
Each facial vascular lesion requires careful clinical, surgical, and radiological evaluation. Facial asymmetry and disfigurement were major sources of discomfort for the patient (**A**,**B**). Moreover, recurrent trauma to the lower lip during mastication led to intermittent bleeding episodes, further aggravating his symptoms (**B**,**C**). Secondly, treatment aimed to relieve teeth-related pain and remove retained roots embedded within the vascular tissue that were contributing to recurrent inflammation (**D**). The main lesion occupied the submandibular, mandibular, cheek, chin, and intraoral regions, with a maximum dimension of 7.39 × 6.78 cm.

A 72-year-old man was referred for management of a severe odontogenic infection associated with an extensive facial vascular lesion. The patient’s medical history was significant for arterial hypertension and chronic liver dysfunction (CLD) of unclear etiology. These factors were noted as a high-risk factor for intraoperative and post-operative bleeding. Given the patient’s CLD, both a local internal medicine specialist and the patient’s hepatologist were consulted preoperatively. Complete blood testing, including coagulation assessment and liver ultrasonography, were performed, with no contraindication to surgery identified. In preparation for surgery, blood units and plasma were prepared just before the surgery. He had been diagnosed many years earlier with a large vascular malformation involving the left mandibular and facial region, which had been managed conservatively with intermittent sclerotherapy using polidocanol (Aethoxysklerol^®^ 2%; lauromacrogol 400, Kreussler, Wiesbaden, Germany) (AE). Clinical examination revealed marked left-sided facial asymmetry involving the submandibular, mandibular, cheek, chin, and intraoral regions. The lesion extended into the mandibular bone and adjacent soft tissues, including skin, oral mucosa, and facial musculature. Intraoral examination demonstrated poor oral hygiene, multiple retained roots, advanced carious lesions, periodontal disease, and signs of chronic odontogenic infection (Figure 2 and Figure 3). From a dental perspective, the patient presented not only with multiple infectious foci but also with a functionally non-maintainable oral condition. Poor oral hygiene, advanced crown destruction, retained roots, periodontal involvement, and traumatic contact with adjacent soft tissues contributed to recurrent pain, local irritation, and difficulty with chewing and oral self-care. Because even routine dental procedures carried a substantial hemorrhagic risk in the setting of the vascular lesion, the patient remained in a cycle in which dental disease progressed while conventional outpatient treatment options remained severely limited. Although the overlying skin was not discolored, a warm, pulsatile submucosal swelling was noted (Figure 3B,C), associated with mucosal irritation caused by traumatic occlusion.

Radiological assessment included panoramic radiography and contrast-enhanced computed tomography (CT) with angiographic sequences. Imaging was used to evaluate the extent of the vascular lesion, its relationship with surrounding anatomical structures, osseous involvement of the mandible, and the presence of odontogenic cysts and periapical pathology. These investigations confirmed a large, complex vascular lesion consistent with a mixed vascular etiology involving both intraosseous and soft tissue components (Figure 3). The lesion demonstrated slow but progressive enlargement over time, with the increasing involvement of adjacent structures, functional impairment of the left oral commissure, and recurrent inflammatory changes related to advanced dental disease. Conventional local dental treatment was avoided because of the extensive vascular lesion, the substantial risk of bleeding, and the lack of a safe treatment plan that could meaningfully improve the patient’s overall well-being. The main concerns related to the lesion were not limited to facial disfigurement and poor esthetics, but also included the patient’s willingness to undergo treatment aimed at improving his oral condition, restoring more effective biting and chewing, relieving pain (primarily tooth- and sinus-related rather than attributable to the mixed hemangioma itself; therefore, no VAS pain score for the lesion was recorded), controlling infection and bleeding risk, and enabling subsequent prosthodontic rehabilitation. The patient was unwilling to undergo major resection with microsurgical flap reconstruction; therefore, a less radical approach was planned. Given the high hemorrhagic risk, conventional dental treatment was contraindicated. A staged treatment plan was therefore chosen with the following aims: (i) to reduce hemorrhagic risk and eliminate active odontogenic foci; (ii) to convert the oral cavity from an infection-prone and functionally compromised state into a safer, more maintainable environment; and (iii) to improve daily oral function, including pain-free mastication, hygiene access, and future prosthetic feasibility, while also reducing facial asymmetry and inflammation-related discomfort.

The first stage consisted of preoperative sclerotherapy using AE (20 mg/mL) administered intra- and extra-orally at five sites (2 mL per ampule) over two sessions, one and two months prior to surgery, to reduce vascular flow and bleeding risk. Comprehensive anesthesiology and internal medicine evaluations were performed before surgical intervention. Given the extent of the lesion, palliation rather than complete eradication was considered the safest and most realistic strategy. Because of the worsening dental condition, ongoing infection, and the patient’s wish to address both the dental disease and the facial asymmetry caused by the hemangioma, he was referred to our department. His clinical presentation suggested facial inflammation, swelling, and local discomfort secondary to dental infection. The lesion itself had been present for many years, resulting in slow growth over time, causing visible facial disfigurement and the decreased possibility for any dental treatment because of the dental risk of some serious and possibly life-threatening bleeding incidents. The patient’s dental needs included the removal of two odontogenic cysts located in the right and central regions of the mandible, together with the extraction of the involved teeth. These lesions were associated with local pain, inflammation, and ongoing clinical problems. The progression of odontogenic inflammation to abscess formation may pose a serious risk to the patient. The so-called odontogenic infections (OIs) need a lot of attention and possible treatment to avoid any inflammatory and purulent manifestation or the spread of local oral infections to adjacent anatomical areas. Each vascular lesion placed in the mandibular bone (Figure 2, red arrow), as well as in the surrounding soft tissues involving skin, mucosa, and muscle, requires some special attention. A thorough intraoral examination, complemented by CT or CBCT imaging, may help define the patient’s dental and surgical needs. The patient’s extensive VM was the main source of decreased oral hygiene and possibility to conduct any suitable dental treatment.

**Figure 4:**
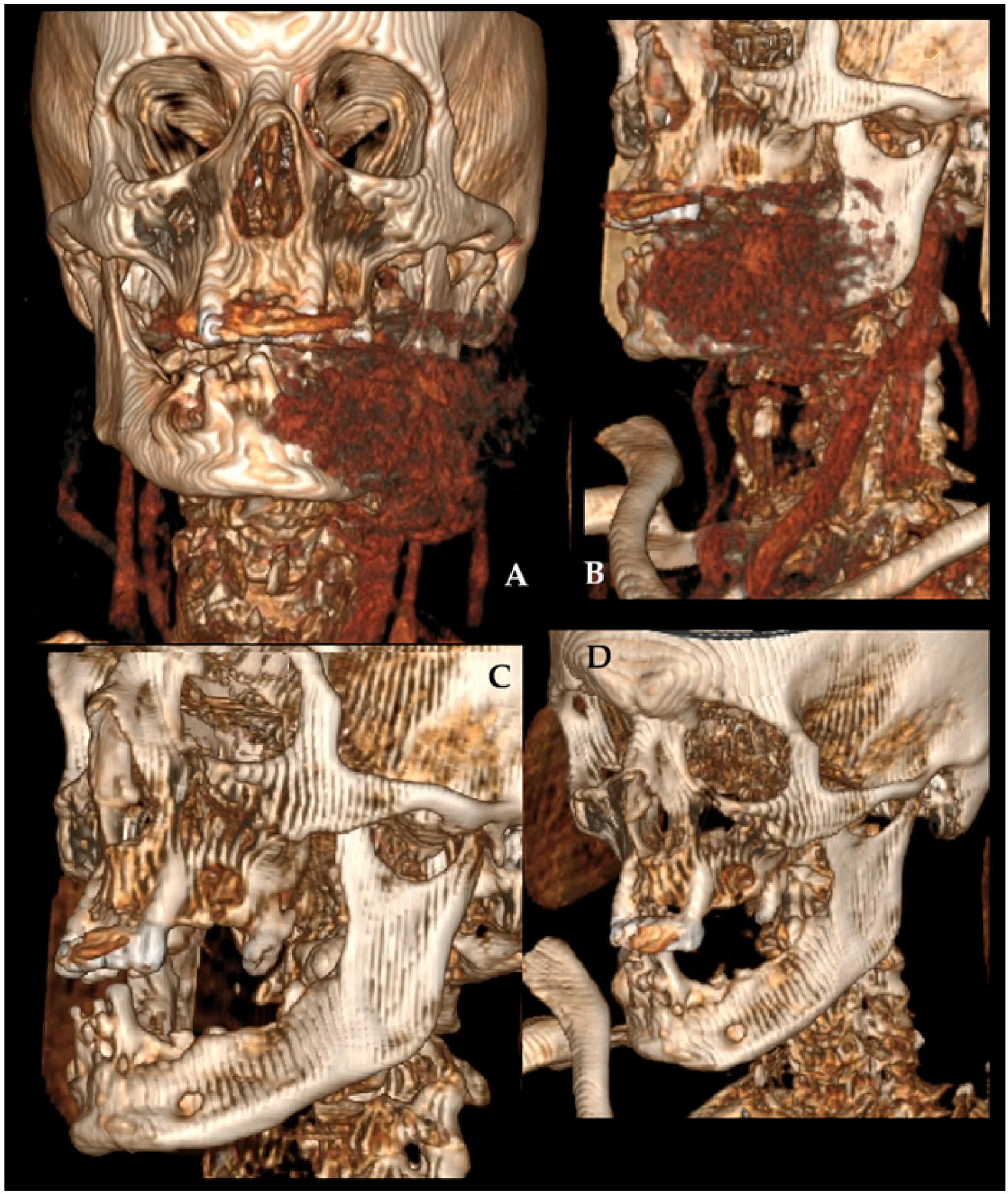
Contrast-enhanced CT and angio-CT imaging demonstrating the extent of the facial vascular malformation and its relationship to surrounding hard and soft tissues. The lesion involves the left submandibular, mandibular, buccal, chin, and intraoral regions, with extension into the mandibular corpus and adjacent soft tissues. Imaging also helped identify associated odontogenic pathology and supported evaluation of hemorrhagic risk before treatment planning (**A**–**D**).

To address the patient’s needs, including the elimination of all odontogenic infectious foci within the oral cavity and palliative excision of the vascular lesion, a two-stage treatment approach was planned. First, 2% AE was administered intraorally and extraorally at one and two months before surgery to induce a local therapeutic effect (Figure 3). During preparation for surgery, the patient underwent the necessary anesthesiologist and internal medicine consultation. Based on the Mulliken and Young classification the vascular lesions (VLs) can be diagnosed into hemangiomas (most common for early infancy) and VMs that are quite common in the maxillofacial area in different forms (slow-flow, fast-flow or mixed type with subtypes) [10,11,12,14,22]. Those maxilla–mandibular lesions might have unpredictable outcomes, growth, location and patterns, and therefore extensive lesions should be treated by specialized facilities in this field of VL. These vascular lesions may be life-threatening because of their potential for spontaneous bleeding, progressive enlargement, and severe hemorrhage. Such events may occur spontaneously or be triggered by tooth extraction, local anesthetic injection, or other dental procedures, potentially resulting in life-threatening bleeding complications. Possible approaches might include surgical and non-surgical means, like local electrocoagulation, injection therapy with sclerosing agents and solutions, intravascular embolization, or resection composed of conservative, radical or palliative approaches, as suggested by some authors [10,11,12,14,16,18,22]. Figure 3 and Figure 4 present the scope of MH as presented in angio-CT, which was evaluated by two independent radiologists and concluded to be a slow-flow lesion, with its spread and complex vascular system surrounding the facial and submental arteries, without any spread towards major neck arteries.

The lesion involved the skin and subcutaneous tissues of the oral commissure, lower lip, and buccal region, as well as the oral mucosa, buccal musculature, surrounding soft tissues, and the left mandibular corpus. The extent of involvement resulted in marked asymmetry of the lower left facial region. Although the overlying skin showed no discoloration, a warm, pulsatile submucosal swelling with focal erythema was observed, accompanied by local irritation secondary to traumatic dental contact. The lesion had shown slow progressive enlargement over time, with the increasing involvement of surrounding structures, causing local pain, discomfort, a reduced function of the left oral commissure, and recurrent inflammatory changes in the adjacent oral mucosa associated with advanced carious teeth. The therapeutic goals are to improve the patient’s quality of life, reduce bleeding risk, eliminate sources of dental inflammation, and restore more effective chewing and biting. In many advanced cases, the primary aim is to improve local well-being and reduce distressing symptoms rather than to achieve complete lesion resection and definitive cure. Surgery is a commonly employed treatment modality in cases such as the one presented here. Adjunctive sclerotherapy, for example with bleomycin, may help reduce the extent of resection and improve overall treatment outcomes.

**Figure 5:**
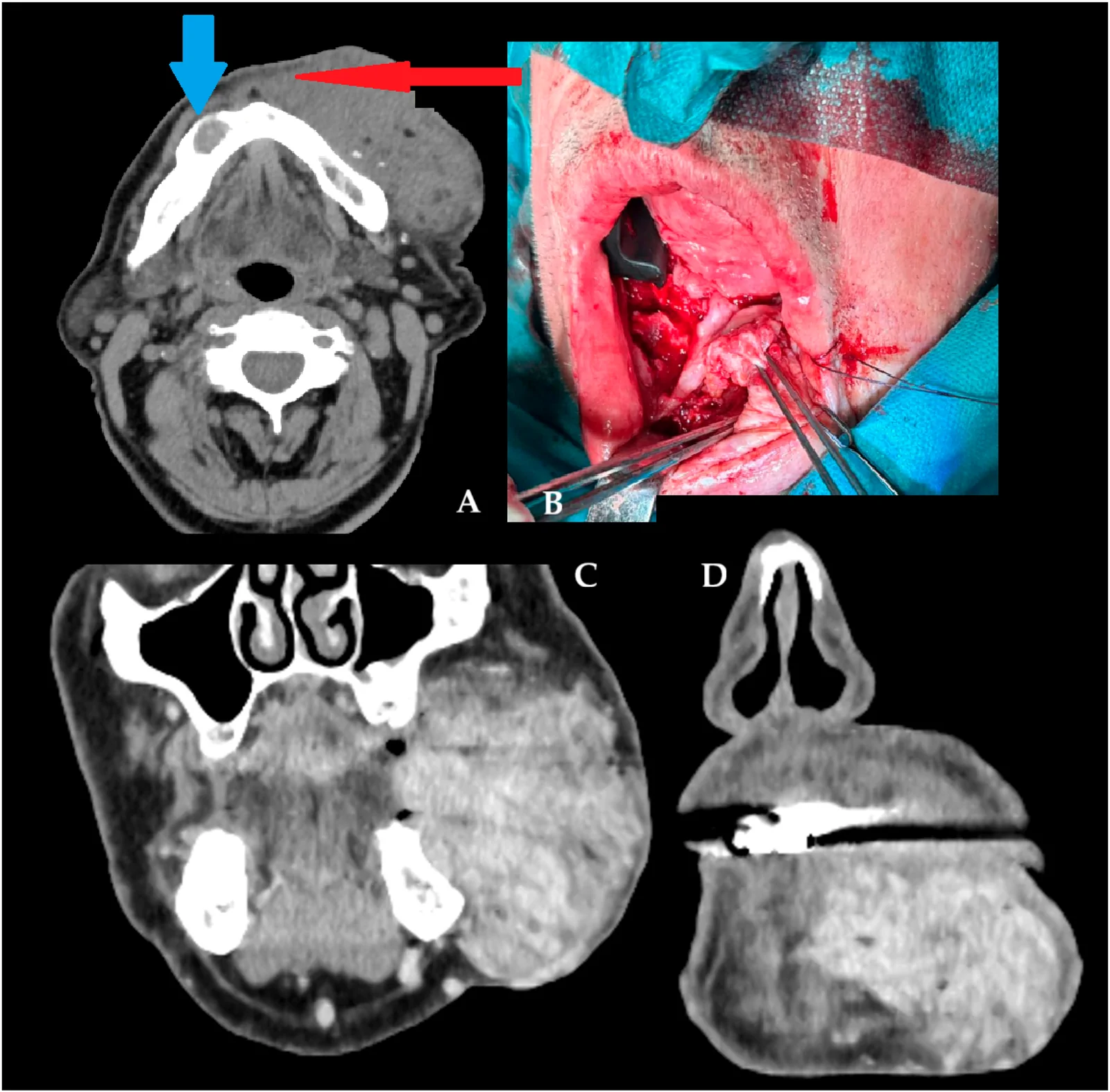
The extent of the presented facial MH indicated that complete treatment and definitive cure were not achievable in this case. Congenital vascular lesions may show variable anatomical distribution and growth patterns. The blue arrow indicates an odontogenic cyst, while the red arrow highlights the mixed vascular component of the hemangioma (**A**). Therefore, a combined staged approach consisting of sclerotherapy followed by palliative surgical excision was performed to reduce lesion volume, minimize bleeding risk, and alleviate symptoms (**B**–**D**).

The goal of treatment for each lesion is to improve the patient’s quality of life and reduce troublesome symptoms and clinical complications (Figure 4 and Figure 5). This case illustrates how a combined approach of sclerotherapy and resection improved the patient’s oral condition, enabled the removal of infected teeth and subsequent dental treatment, enhanced oral hygiene, and created the basis for future prosthetic rehabilitation aimed at restoring bite, occlusion, and chewing function. Another therapeutic goal was to alleviate facial asymmetry, cosmetic and functional impairment, painful swelling, bleeding episodes, infectious foci, and the risk of abscess formation, while promoting the resolution of skin and mucosal lesions and elimination of teeth-related pathology. The application of sclerosing agents before each surgical approach to vascular lesion therapy also seems a good first-step approach to reducing bleeding during the surgical excision. The alternative is a vascular embolization in selected cases. Therapeutic management is often individualized according to the specific features of each case, as well as the surgeon’s experience and preferences in treating such lesions. In cases of diffuse facial vascular lesions associated with multiple direct and indirect feeding vessels, the treatment approach requires meticulous surgical planning. Some authors also recommend prophylactic embolization or sclerotherapy to limit further angiogenesis [10,11,12,13,14,15]. While certain intraosseous vascular lesions may be treated with surgery alone, together with management of the involved teeth in the adjacent bone, combined venous, arterial, and mixed lesions more often require combined treatment approaches. In the presented case, in order to improve the patient’s quality of life, the authors performed a two-step surgical approach. First, a sclerotherapy with 2% AE was used for five different areas as a preparation for therapy (total 2 mL of AE solution mixed with 5 cm of foam, guided without USG, based on the most prominent MH site). After four weeks, the patient was scheduled for a palliative excision of the vascular facial lesion with teeth removal, local bone debridement under general anesthesia and naso-tracheal intubation. An extraoral submandibular incision was made just below the vascular lesion, with preservation of the marginal mandibular branch of the facial nerve. Surgical maneuvers were achieved by the usage of:

1. “*ligatura-intra-tumorale*”, 2.0 Ethibond haemostathic suturing (HS).
2. Aethoxysklerol (2% solution, lauromakrogol 400—polidocanol, Kreussler, Wiesbaden, Germany) injections within other smaller peripheral lesions, because of the scope of this palliative surgery (1 ampule, 2 mL AE solution).
3. Spongostan (haemostathic sponge, Medisponge/Surgispon 7 × 5 × 1 cm, ProMedica, Gorzów Wielkopolski, Poland) packed in the bone lesion and empty bone cavities.
4. Surgicel (S) (cellulose hemostatic dressing, Surgicel Original 5 × 1.25 cm, Johnson & Johnson, Somerville, NJ, USA) to promote hemostasis in the perimandibular area.
5. Tachosil (hemostatic dressing, Fibrynogen 5.5 mg Trombin 2.0 j.m. Takeda, Osaka, Japan) used for compressive dressing with mixture of HS and S.
6. Clips and ligation (polymer clips, Grena Click’aV, Grena Ltd., Chelsea, UK) used to maintain bleeding for other bigger vessels.
7. Bone wax (Ethicon a’12, Johnson & Johnson, Somerville, NJ, USA) to fill the bone defects and additional bleeding bone areas after bone revision, debridement and ostectomy.
8. Coagulation—typical coagulation and termoablation of adjacent structures.
9. Compression dressing in the submandibular area combined with Codofix dressing and elastic bandage.
10. Redon drainage in the submental area and retromandibular area.
11. Preparation of four units of blood and six units of plasma were prepared in case of extensive bleeding; however, none were used during operation.

Additional intravenous agents were administered one hour before and after surgery and were continued for the next 5 days. It consisted of: Exacyl (tranexamic acid, 1 g 2× per day, solution: 100 mg/mL (500 mg/5 mL); 5 amp. 5 mL—Cheplapharm, Greifswald, Germany) with Cyclonamine (etamsylatum, 500 mg four times per day, 12.5%, 125 mg/mL, 2 mL, Galena, Wrocław, Poland). Those additional hemostatic agents were important because of advanced VM and the scope of an undiagnosed liver condition that might also affect prolonged or delayed bleeding occurrence. Total blood loss was approximately 1 L, whereas higher intraoperative blood loss of up to 2 L had been anticipated; therefore, multiple blood units were prepared preoperatively. For the following reason the patient did not require any blood transfusion, because the patient overall was in a good condition after surgery, with a stable heart rate and blood pressure. Typical balanced intravenous drips were used. Perhaps a lot of AE therapy before surgery, combined with step-by-step dissection with a lot of surgical maneuvers listed above, could have affected the final result.

**Figure 6:**
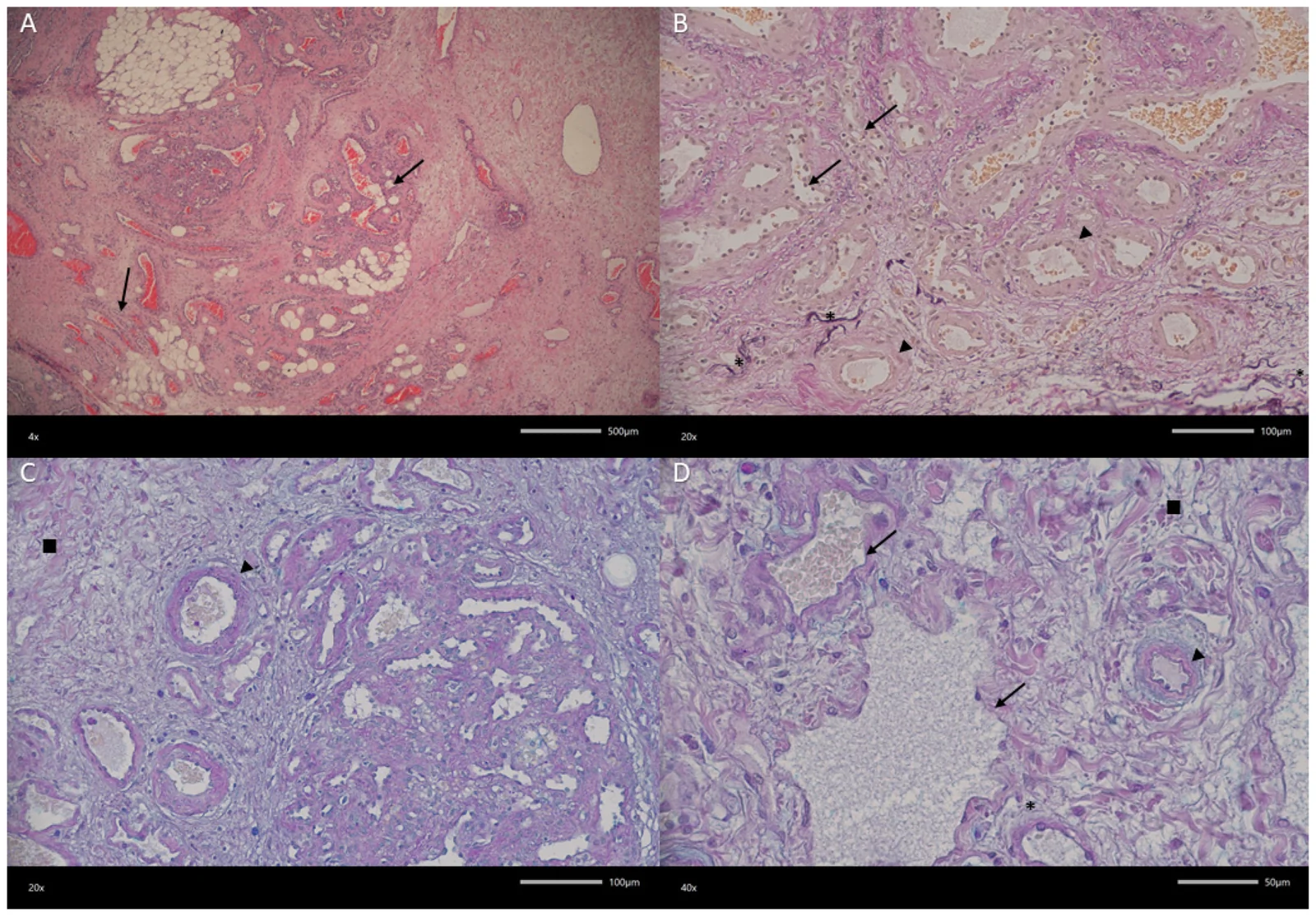
Histological examination confirms the diagnosis of mixed-type hemangioma (MH). Representative sections demonstrate vascular structures of varying caliber and architecture, consistent with a mixed structure (triangle, square and arrow caption) (**A**–**D**). The asterix (*) is a capillary vessel supported by elastic fibers.

Samples were fixed in 4% formaldehyde buffered solution, pH 7.2–7.4, for 48 h then washed in tap water (Figure 6). After washing for 24 h, the material was dehydrated in alcohol series and embedded in paraffin. Sections 7 µm thick were stained with hematoxylin and eosin, Elastica van Gieson Kit, Alcian blue/PAS staining and analyzed in a Nikon Eclipse 80i microscope (Nikon Corporation, Tokyo, Japan). The results include: In the examined samples, numerous blood vessels of varying architecture and caliber, surrounded by a fibrous capsule, are observed (Figure 6A). Regions containing densely packed capillaries filled with erythrocytes and lined by flattened endothelial cells are evident. Larger vessels, predominantly arterioles (arrowheads), are located at the periphery of the lesion. Within these arterioles, sparse elastic fibers are present (Figure 6B). Between the capillaries, only a minimal amount of fine, loosely arranged collagen fibers can be identified, and no elastic fibers are visible in these areas. Additionally, large, dilated vascular spaces filled with blood are noted, their thin walls separated by loose fibrous stroma (Figure 6C,D). These histological features suggest that the lesion represents a mixed-type hemangioma (MH). A—Overall structure of biopsy. Visible bundles of mixed vessels (black arrow) in the connective tissue separated by fibrous capsula. B—Numerous capillaries surrounded by arteriole (black arrow). Single elastic fibers are mostly found in the walls of larger vessels (asterisks). C—The collagen fibers (black square) are visible exclusively in capsula and intima of larger vessels. D—Very thin expanded vessels contain very thin walls supported by very thin fibers. Mag. A—40×, B,C—200×, D—400×, A—hematoxylin and eosin staining, B—Elastica van Gieson staining kit, C,D—Alcian blue/PAS staining.

**Figure 7:**
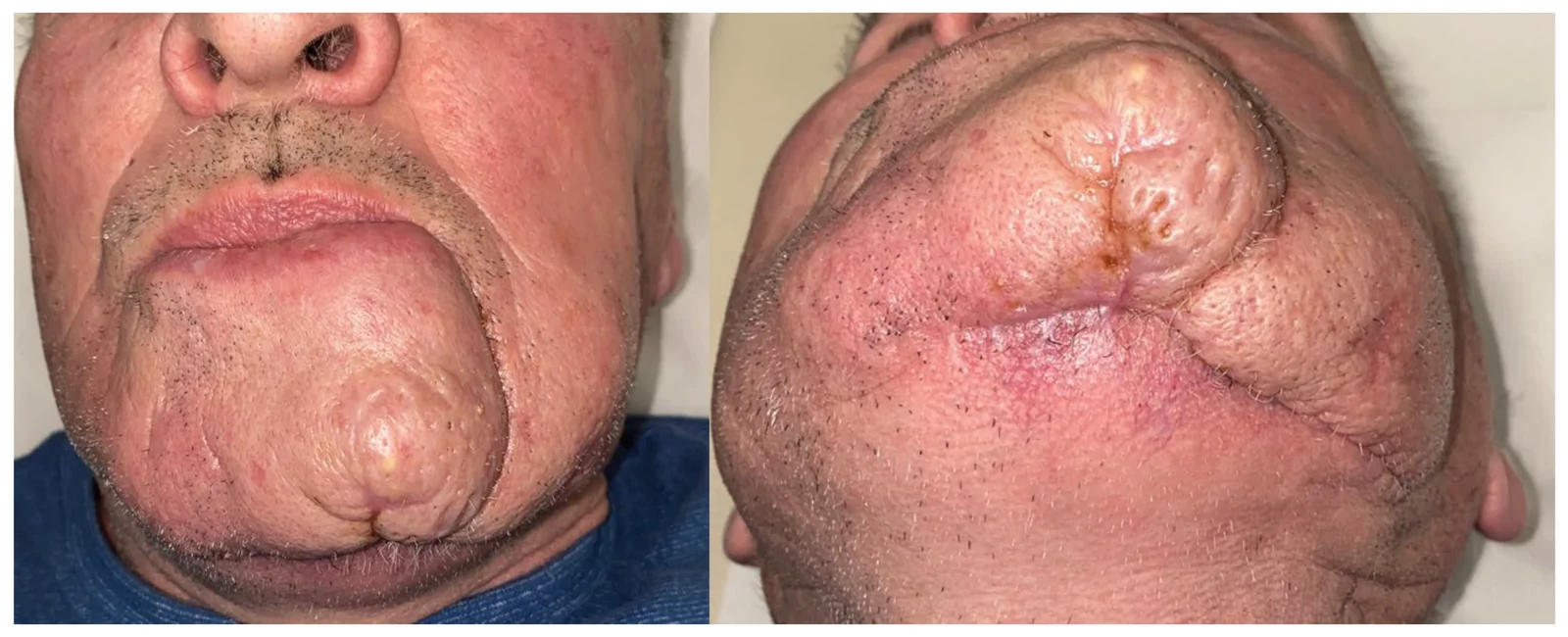
Both the immediate postoperative outcome and the 12-month follow-up showed improved facial symmetry and absence of worrisome bleeding. A final corrective procedure was planned to reduce excess skin tissue and perform local plastic surgery to improve facial contour and scar appearance.

The staged therapeutic approach was completed without intraoperative or immediate postoperative complications. No episodes of uncontrolled bleeding occurred during surgery or in the early postoperative period. Wound healing progressed uneventfully, with no evidence of infection or wound dehiscence. Following treatment, the patient reported a marked relief of pain and discomfort. Facial swelling and asymmetry were substantially reduced, and mucosal irritation caused by traumatic occlusion resolved completely. Oral hygiene improved, and the patient regained satisfactory mastication and speech without major functional limitation. These postoperative benefits were assessed clinically and through patient-reported improvement rather than with standardized quality-of-life instruments. Postoperative imaging confirmed partial removal and volume reduction in the vascular lesion together with the complete elimination of intraosseous odontogenic pathology. No signs of mandibular osteonecrosis or pathological fracture were observed. At six-month follow-up, the patient remained free of recurrent odontogenic infection, abscess formation, or spontaneous bleeding episodes (Figure 7). No progression of the residual vascular lesion was noted. Several months later, the patient died due to previously undiagnosed hepatocellular carcinoma unrelated to the maxillofacial pathology described in this report. This case report was prepared in accordance with the CARE case report guidelines.

## 3. Discussion

From a therapeutic perspective, the management of vascular anomalies of the craniofacial region encompasses a broad spectrum of interventions ranging from conservative monitoring to complex multimodal therapy. Contemporary protocols emphasize individualized treatment selection based on flow characteristics, anatomical extent, symptom severity, and hemorrhagic risk, often combining sclerotherapy, embolization, laser therapy, and staged surgical excision to achieve optimal outcomes [23,24]. Recent reports indicate that combined approaches provide superior long-term control compared with single-modality treatment, particularly in diffuse or mixed lesions involving bone and soft tissues [25,26]. Furthermore, preoperative devascularization techniques are increasingly regarded as essential adjuncts for minimizing intraoperative blood loss and improving surgical safety in high-risk VMs [27].

The uniqueness of this case highlights the need for a full-mouth therapy (FMT) addressing the removal of all dental sources of inflammation leading to the formation of local gum swelling, jaw pain and irritations, and decreasing the risk of any abscesses within the proximity of any VMs. Furthermore, despite most of the known presented vascular studies emphasizing the possibilities and methods of each VM treatment, only some of them fully address the patient’s need for an oral cavity free of teeth-related pain and swelling, and showing improved local oral cavity status. A single tooth removal or any periodontal procedure in the proximity of VM might lead to serious life-threatening bleeding, which local dental practices are not always prepared to handle adequately.

The published literature on facial vascular anomalies has greatly focused on lesion classification, imaging work up, and lesion-directed treatment strategies such as embolization, sclerotherapy, and staged resection, with comparatively limited attention to the oral infectious burden as an independent determinant of risk and treatment planning [10,16,23,24,25,26,27,28]. Reviews and larger case series consistently stress the importance of multidisciplinary management and individualized treatment in head and neck vascular malformations, especially when both bone and soft tissue are involved [7,16,17,23,29,30,31,32,33,34,35]. However, they rarely address how advanced dental disease can itself trap patients in a cycle of pain, inflammation, poor oral hygiene, and delayed care, as even routine dental treatment may carry a substantial risk of major hemorrhage [23,29,30,31,32,33,34,35,36]. Likewise, many published dental case reports as well as similar papers have centered on abundant bleeding during or after the extraction of previously unrecognized mandibular vascular malformations, thereby highlighting diagnostic danger rather than planned oral rehabilitation [36,37,38,39,40,41,42,43,44,45,46,47,48,49,50]. More recent reports addressing dental treatment in patients with known extensive vascular malformations have generally focused on hospital-based extraction of a single tooth or on localized oral surgical interventions [49]. By contrast, the clinical value of the present case lies in illustrating a broader treatment strategy. Rather than focusing solely on the safe management of a vascular lesion, this case demonstrates a staged approach to improving a chronically infected and functionally compromised oral cavity. This was achieved through lesion-directed preoperative preparation, the simultaneous elimination of multiple odontogenic infection foci, and palliative surgery aimed at improving mastication, access for oral hygiene, pain control, and the feasibility of future prosthetic rehabilitation. Therefore, the novelty of this report is not based on the rarity of vascular malformation alone, but on the underreported intersection of an extensive facial vascular anomaly, multifocal odontogenic disease, hemorrhagic risk, and patient-centered functional rehabilitation within a single coordinated treatment strategy.

The management of extensive facial vascular anomalies remains one of the most challenging areas in oral and maxillofacial surgery. These lesions often exhibit unpredictable growth, complex vascular architecture, and a substantial risk of hemorrhage, particularly when invasive procedures are required. The present case illustrates the additional complexity introduced by severe odontogenic infection in a patient with a large MH involving both soft tissues and the mandibular bone. An important aspect of the present case is that the dental disease was not merely an incidental finding but a major determinant of risk, symptoms, and treatment limitation. In extensive facial vascular anomalies, chronic odontogenic pathology may create a persistent inflammatory background that worsens pain, mucosal irritation, chewing inefficiency, and hygiene neglect, while a fear of provoking hemorrhage often delays definitive dental care. This combination may gradually produce a “dentally trapped patient,” in whom oral disease progresses despite the clear need for intervention. Therefore, dental assessment in such cases should be viewed not only as infection screening, but also as a functional and quality-of-life evaluation. In the presence of vascular anomalies, odontogenic infections may precipitate severe complications. Local inflammation increases tissue perfusion and vascular fragility, while abscess formation near VMs may trigger uncontrollable bleeding. Consequently, even routine dental procedures can become life-threatening or lead to unwanted local events [15,16,17,18,19,21,28,29]. It is quite advisable to assess the condition of the entire oral cavity clinically and in CBCT soon before any surgical or vascular procedure, in order to schedule any necessary dental treatment simultaneously during the major surgery that can be beneficial for the patient in terms of improved oral cavity status. Dental therapy is often underestimated in patients with VMs, although it may substantially improve the local condition and quality of life.

While most radiological assessments are focused on the main lesion, teeth and the status of teeth-bearing structures are somehow quite commonly forgotten and underestimated. Not only should dentists assess each patient, but a laryngologist should also examine the sinuses as well as the nasal cavity, while an ophthalmologist should assess any possible spread to the eye socket. The teeth’s relationship with sinus and nasal-related diseases, as well as those affecting the eye socket, should be remembered. As a result, the importance of dental control in any possible teeth-related infection or assessment of a patient’s oral needs is important [16,17,18,19,21,39,40,41,42,43,44].

Odontogenic infections, particularly when chronic and untreated, represent a persistent inflammatory burden that may significantly aggravate adjacent vascular anomalies. The close anatomical relationship between posterior maxillary teeth and the maxillary sinus explains why odontogenic sinusitis frequently develops secondary to periapical pathology, retained roots, or periodontal disease, often presenting with unilateral symptoms and radiographic opacification [30,31]. In patients with coexisting vascular malformations, inflammatory hyperemia, local cytokine release, and increased tissue pressure may further compromise vascular stability, potentially precipitating bleeding, thrombosis, or rapid lesion expansion [32,33]. Therefore, the early radiological identification of odontogenic sinusitis using panoramic imaging, CBCT, or CT, followed by a definitive elimination of the dental source, should be considered an essential component of risk reduction in complex craniofacial vascular cases [34].

The staged approach adopted in this case aligns with contemporary management principles for complex craniofacial vascular anomalies, in which therapeutic success is defined not solely by lesion eradication but by functional rehabilitation and risk reduction. Preoperative sclerotherapy enabled a safer surgical intervention by decreasing vascular flow, while palliative excision addressed infection foci and mechanical dysfunction without exposing the patient to excessive operative morbidity [10,11,12,13,14,15,16,17,18,19]. Rather than prioritizing radical resection, the treatment focused on the restoration of oral homeostasis, elimination of inflammatory triggers, and stabilization of local tissues, which are increasingly recognized as key determinants of long-term clinical stability. Current evidence suggests that outcomes in patients with head and neck VMs should be assessed through multidimensional endpoints including functional capacity, symptom burden, and psychosocial well-being rather than an anatomical resolution alone [7,20,35,36,37]. In this context, the observed improvement in mastication, hygiene, comfort, and social confidence highlights the importance of individualized, goal-directed therapy strategies tailored to patient needs and lesion biology. Although standardized oral health-related quality-of-life questionnaires were not applied in this case, the clinical course strongly suggests a meaningful patient-centered benefit. The relief of chronic teeth-related pain, reduction in mucosal trauma and intermittent bleeding, improvement in oral hygiene, and restoration of more comfortable mastication likely contributed substantially to daily well-being. In patients with visible craniofacial vascular lesions, such gains may be particularly relevant because oral discomfort, poor dentition, and facial deformity often interact to amplify both functional limitation and psychosocial distress. Such functional gains are clinically meaningful because even moderate improvements in oral competence, pain reduction, and facial symmetry can substantially enhance nutritional status, speech intelligibility, and interpersonal interaction, particularly in elderly patients with chronic comorbidities [22,38]. Importantly, the relief of persistent odontogenic inflammation may also reduce systemic inflammatory burden and fatigue, factors increasingly associated with diminished overall health perception and reduced daily activity levels [39,40]. From a psychosocial perspective, visible craniofacial lesions often carry a disproportionate emotional impact, influencing self-esteem, social participation, and treatment acceptance; therefore, therapeutic strategies that visibly improve facial balance and eliminate recurrent symptoms may yield benefits extending well beyond the surgical field [41,42]. These findings support the view that the management of complex vascular anomalies should prioritize patient-reported outcomes and functional improvement, rather than focusing exclusively on radiographic changes [43]. A typical tooth pain caused by local inflammation might cause not only a severe bleed when positioned in an AVM, as reported by Kriwalsky et al., but also untreated might form a submucosal abscess that spreads further [44]. Life-threatening complications may follow tooth extraction, especially when immediate hemorrhage originates from an underlying oral vascular lesion or when progressive swelling results in restricted mouth opening, trismus, and potential airway compromise requiring urgent life-saving management [45]. On the other hand inflammatory induced trismus, cause by some dental infections, might lead to compromised airways, and therefore it is essential to approach each patient with VMs through a team approach including the status of the skin, teeth, sinuses and even eye-sight. Both Engel et al. and Petronis et al. reported that dental pathology may cause various teeth-related complications and infections, although their severity and progression may take time to evolve into a serious clinical problem [45,46]. In those cases, a detailed and improved dental examination should always consist of clinical and radiological examination with a CBCT.

A review by Pascual-Castroviejo and Pascual-Pascual concluded that, while some congenital or vascular lesions, marks or anomalies present after birth might be locally controlled, for example with propranolol intraoral uptake, some other lesions present, growing and progressing at different times, might pose a challenge to treatment and for patient prognosis [47]. On the other hand, a review by Carqueja et al. emphasizes that each vascular lesion requires a strict multidisciplinary team and collaboration in order to focus not only on the vascular lesion itself, but also on patient airways, bite, occlusion, quality of life and also dentition and the related sources of infection [48]. Each case may present differently and require a tailored approach, although the common goal remains the improvement of the patient’s local and general condition. The following facts emphasize the author’s philosophy of an FMT and collaboration between different medical specialists in order to achieve, together in the team approach, a more desirable effect for the patient.

The histopathological findings confirmed the mixed vascular nature of the lesion, explaining its long-standing behavior and resistance to complete surgical cure. In such cases, long-term follow-up remains essential because of the potential risk of progression or recurrence. This report is limited by its single-case design and by the absence of standardized quality-of-life or pain assessment tools. Owing to the patient’s fragile general condition, the complexity of the vascular lesion, and the need to prioritize infection control and bleeding risk reduction, postoperative improvement was assessed mainly through clinical findings and patient-reported functional benefit. In addition, the patient’s later death from unrelated hepatocellular carcinoma prevented longer-term formal quality-of-life evaluation. Nevertheless, the case highlights a clinically important and underreported scenario with relevant implications for patient safety.

Future studies should aim to establish standardized diagnostic and treatment algorithms integrating dental, radiological, and vascular assessments to optimize safety in patients with complex craniofacial vascular lesions. Prospective multicenter analyses would be particularly valuable to determine predictive factors for complications and to refine indications for staged or palliative approaches. Additionally, the incorporation of validated quality-of-life instruments and patient-reported outcome measures may provide a more objective evaluation of therapeutic success and facilitate comparison between treatment modalities. Such data could contribute to evidence-based guidelines for interdisciplinary management of these rare but clinically demanding conditions.

Study limitations might include a missing questionnaire to assess quality of life before and after surgery, as well a questionnaire to assess oral status and dental needs before and after treatment, but those two facts will definitely be studied in future studies, if a larger study group of patients with the same vascular anomaly will be treated, to ensure that the results are more precise and professional. In such cases, the main goal should always be focused on a solution to help each patient with their respective condition in the most reasonable way possible.

## 4. Conclusions

Severe odontogenic infection in the presence of extensive facial vascular anomalies represents a high-risk clinical scenario requiring meticulous planning and multidisciplinary management. A staged approach combining sclerotherapy with palliative surgical excision can effectively reduce hemorrhagic risk, eliminate infection, and significantly improve oral function and quality of life. In many advanced cases, rather than prioritizing radical resection, treatment strategies should focus on patient safety, functional rehabilitation, and symptom control. Early dental assessment and proactive management of odontogenic disease are essential components of care in patients with complex maxillofacial vascular anomalies.

## Data Availability

The datasets used and/or analyzed during the current study are available from the corresponding author upon reasonable request.
